# Active vision in freely moving marmosets using head-mounted eye tracking

**DOI:** 10.1073/pnas.2412954122

**Published:** 2025-02-03

**Authors:** Vikram Pal Singh, Jingwen Li, Kana Dawson, Jude F. Mitchell, Cory T. Miller

**Affiliations:** ^a^Department of Psychology, Cortical Systems and Behavior Lab, University of California San Diego, San Diego, CA 92093; ^b^Department of Brain and Cognitive Science, University of Rochester, Rochester, NY 14627; ^c^Department of Psychology, Neurosciences Graduate Program, University of California San Diego, San Diego, CA 92093

**Keywords:** vision, marmosets, ethology, eye-tracking, gaze stabilization

## Abstract

Our understanding of how vision functions as primates actively navigate the real-world is remarkably sparse. As most data have been limited to chaired and typically head-restrained animals, the synergistic interactions of different motor actions/plans inherent to active sensing—e.g., eyes, head, posture, movement, etc.—on visual perception are largely unknown. To address this considerable gap in knowledge, we developed an innovative wireless head-mounted eye-tracking system that performs Chair-free Eye-Recording using Backpack mounted micROcontrollers (CEREBRO) for small mammals, such as marmoset monkeys. Because eye illumination and environment lighting change continuously in natural contexts, we developed a segmentation artificial neural network to perform robust pupil tracking in these conditions. Leveraging this innovative system to investigate active vision, we demonstrate that although freely moving marmosets exhibit frequent compensatory eye movements equivalent to other primates, including humans, the predictability of the visual behavior (gaze) is higher when animals are freely moving relative to when they are head-fixed. Moreover, despite increases in eye/head-motion during locomotion, gaze stabilization remains steady because of an increase in vestibularocular reflex gain during locomotion. These results demonstrate the efficient, dynamic visuo-motor mechanisms and related behaviors that enable stable, high-resolution foveal vision in primates as they explore the natural world.

## Significance

Vision is arguably the most thoroughly understood of all neural systems in the primate brain. Yet little is known about how vision functions in real-world contexts in which individuals freely move and explore an environment. This dearth in knowledge is largely due to the lack of technology that can accurately track eye movements in freely moving individuals with the speed and resolution needed to quantify primate vision. Here, we developed an innovative wireless head-mounted eye-tracking system for marmosets that meets these technical needs and will enable us to study primate vision in a manner not previously possible and make discoveries that are likely to transform our understanding of this keystone system.

Primate vision has been the subject of intense study for many decades and is arguably the most well understood neural system in the simian brain (1, 2). And yet, our understanding of primate vision is incomplete. Like all sensory systems, primate vision evolved in response to the challenges inherent to actively moving, exploring, and engaging with objects, individuals, and the environment from different perspectives (3–5). While the processes of visual encoding have been extensively studied with head-restrained subjects observing stimuli presented on a screen, details of how vision functions as primates actively move through and explore the real-world are remarkably limited. Similar to all vertebrates, primates coordinate their head and eye movements to enable a stable percept of visual inputs (6–10). Previous studies in chaired but head-free macaques demonstrate how the eye and head coordinate to stabilize gaze but have yet to address similar coordination in freely moving primates. Likewise data indicate that the oculomotor range of marmosets is more restricted than macaques when head-fixed (11) and that their head position can shift more rapidly than humans or macaques (12), but we still lack descriptions of the head-eye coordination for gaze shifts in freely moving scenarios. Because data on this complement of mechanisms has been limited to chaired animals unable to locomote, the synergistic effects of different motor actions—e.g., eyes, head, posture, movement, etc.—on primate visual perception and cognition during active exploration of the world are almost entirely unknown. The principal bottleneck has been technical. Several previous studies sought to bridge this gap and achieved limited precision for projecting the gaze of free-moving nonhuman primates (NHPs) into visual scenes (13–15), but due to technical constraints of these methods, no study has been able to quantify the stability of visual gaze nor the eye and head dynamics during freely moving active exploration. Here, we overcome these obstacles and introduce a method that precisely quantifies eye movements and accurately projects the gaze of a NHP into scenes as individuals freely explore an environment.

Recent work with mice demonstrates that eye-tracking systems can be miniaturized and mounted to the head to quantify natural visual behaviors (16–19), but these systems are not well suited for comparable studies in primates for at least two reasons. First, systems in mice rely on a tether which restricts the 3-dimensional (3D) mobility of primates. Second, they lack the precision and temporal resolution needed to accurately characterize high-resolution primate vision. To address this challenge, we developed an innovative head-mounted eye-tracking system to enable the study of active, natural visual behaviors, and related neural processes, in freely moving marmosets. Our system—CEREBRO—allows for Chair-free Eye-Recording using Backpack mounted micROcontrollers at a speed and resolution needed to accurately quantify the visual behavior and underlying neural mechanisms of natural, active vision in primates. Using CEREBRO we confirmed that freely moving marmosets exhibit frequent compensatory eye movements that enable them to stabilize gaze when viewing real world scenes consistent with previous studies in body restrained animals. By using this innovative system, however, we found that visual gaze stabilization and predictability was enhanced when the monkeys were moving naturally despite increases in eye/head-motion during locomotion. This suggests that previously unreported synergistic mechanisms for gaze stabilization are not only integral to primate active vision in the real-world but can be enhanced for greater compensation as animals move naturally through their environment.

## Results

### CEREBRO Is a Head-Mounted Eye-Tracking System for Freely Moving Marmosets.

**Fig. 1.**
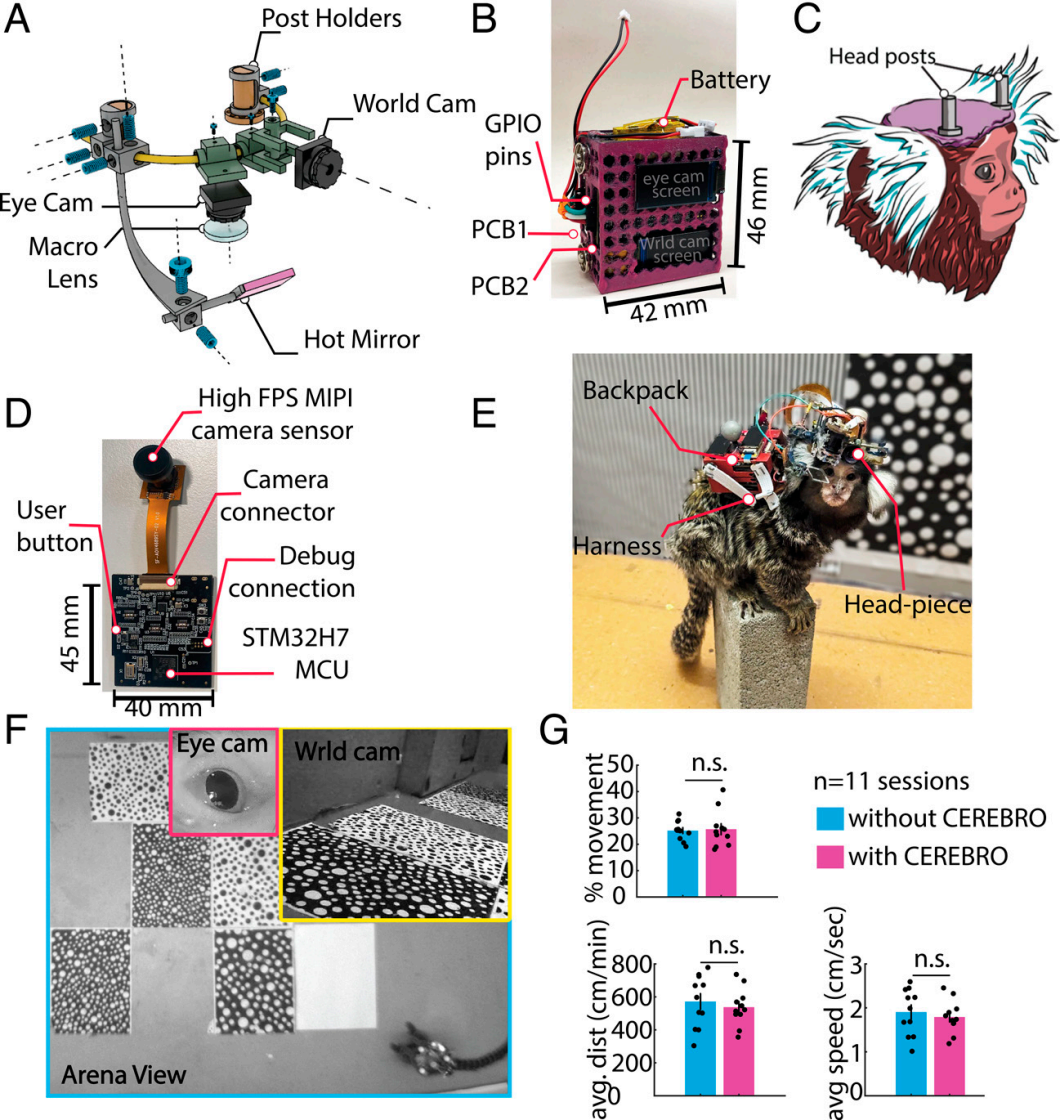
Head-mounted eye-tracker assembly. (*A*) 3D render depicting different parts of the head-piece. The Ti- alloy scaffolding is custom designed and fabricated using Direct-Laser-Sintering (DLS) (*B*) 3D printed backpack that holds the two PCBs and the battery. (*C*) The animals are fitted with two 1 cm headposts that act as anchors for holding the head-piece. (*D*) Custom PCB board which receives data from camera sensors, IMU and saves it to a locally mounted SD card. (*E*) A marmoset wearing the fully assembled system (CEREBRO). (*F*) View from the three different cameras [eye camera (pink), world camera (yellow), and the external arena camera (cyan)] during a freely moving session. (*G*) Animals wearing CEREBRO exhibited no impairment in their free-moving characteristics such as percent movement, distance covered, and speed.

The small body size of marmosets (~300 to 400 g) necessitated certain design considerations when developing CEREBRO. The first of these decisions is related to the weight and wearability of the system itself. Based on previous experience, the estimated 60 g weight of the complete system would not be feasible to be entirely situated on animal’s head without affecting its visual behavior. To resolve this issue, we separated the system into two separate, but integrated hardware submodules: the Head-piece and the Backpack. The Head-piece includes the camera assembly and scaffold fitted to the animals’ head (Fig. 1*A*); and the Backpack includes the backend electronics for camera synchronizing, image acquisition, and local data storage on the custom designed printed circuit boards (PCBs) (Fig. 1*B*). Both the PCB and the head-piece infra-red light-emitting-diode (IR LED) are powered using a 600 mAh, Lithium-Polymer battery that is housed inside the backpack. OV4689 camera modules (SincereFirst, Guangzhou, China) were used to record the eye and the world scene. For more details, see SI Appendix, Supplementary information 1: Camera and Communication Interface.

### Head-Piece Module.

The head-piece weighs ~20 g and comprises five different components (Fig. 1*A*): 1) the scaffold: a curved metal tube that serves as an anchor for all the pieces, 2) eye-cam: an HD MIPI camera (90 fps) with Visible light filter and a Macro lens for looking at the eye, 3) world cam: an HD MIPI camera (60 fps) for capturing the world scene in front of the camera, 4) an IR LED to illuminate the eye, and 5) a strategically placed Hot mirror to image the eye. The mechanical parts for the head-piece are made up of a Titanium alloy (Ti-6Al-4V), and are held to the scaffold using M2 and M4 screw sets (SI Appendix, Supplementary information 2: Detailed Assembly Description). To achieve reliable eye-tracking in a fully unrestrained animal, the eye camera’s position requires to be fixed with respect to the animal’s eye. This is accomplished by attaching two vertical headposts (5 mm diameter, 1 cm tall cylinders with a flat cut on one side) on the animals’ head (Fig. 1*C*). The two headposts restrict any rotation or translational movement of the headpiece and allow the headpiece to be placed at the same position for every recording session.

### Backpack Module.

The backpack module weighs 40 g and comprises two Customized PCBs, as well as the casing and harness worn by the animals (Fig. 1*B*). Each camera has its own dedicated PCB (Fig. 1*D*). Both cameras (eye cam and world cam) have long flex cables (15 cm) and are operated by the PCB.

### Customized PCBs.

The PCB used here is an embedded system that runs on an STM32H750 microprocessor (Fig. 1*D*). The custom PCB consists of a 0.96” Serial-Peripheral -Interface Thin-Film-Transistor (SPI TFT) display to preview camera frames and stores data image stream and Inertial Measurement Unit (IMU) data on a Secure Digital (SD) card. For more details of the PCB and data storage logic, see SI Appendix, Supplementary information 3: Printed Circuit Board and Data Storage.

### Eye-Tracking System Wearability.

CEREBRO allows a full range of natural motor movements when fully configured (Fig. 1*E*), while monitoring its position in the environment, eye position, and view of the scene (Fig. 1*F*). Overall, CEREBRO weighs ~60 g (headpiece module: 20 g + backpack module: 40 g), which is comparable to the weight of two infant marmosets that an adult would normally carry on its back. The habituation protocol for adjusting the animals to carrying the backpack and the head-piece is discussed in SI Appendix, Supplementary information 4: Habituation of Animals to Backpack. To test the influence of this added weight on mobility, we compared marmosets’ behavior both with and without CEREBRO in multiple sessions (30 min) of freely moving exploration in a large open arena (200 cm × 100 cm × 240 cm). The animals were tracked using Optitrack imaging systems. Using the velocity threshold of 5 cm/s, we quantified the percentage of time animals spend moving through the arena. Over 11 sessions, we observed no significant difference (Fig. 1*G*) in the time subjects’ spent locomoting vs. stationary and scanning the environment (*P* = 0.83). We also did not observe any significant difference in either the distance covered by the animals (*P* = 0.569) or the average speed of the animal with and without CEREBRO (*P* = 0.5692). These results suggest that our system does not significantly impede the movements of a freely behaving marmoset.

### Fast, Efficient, and Accurate Pupil Detection.

**Fig. 2.**
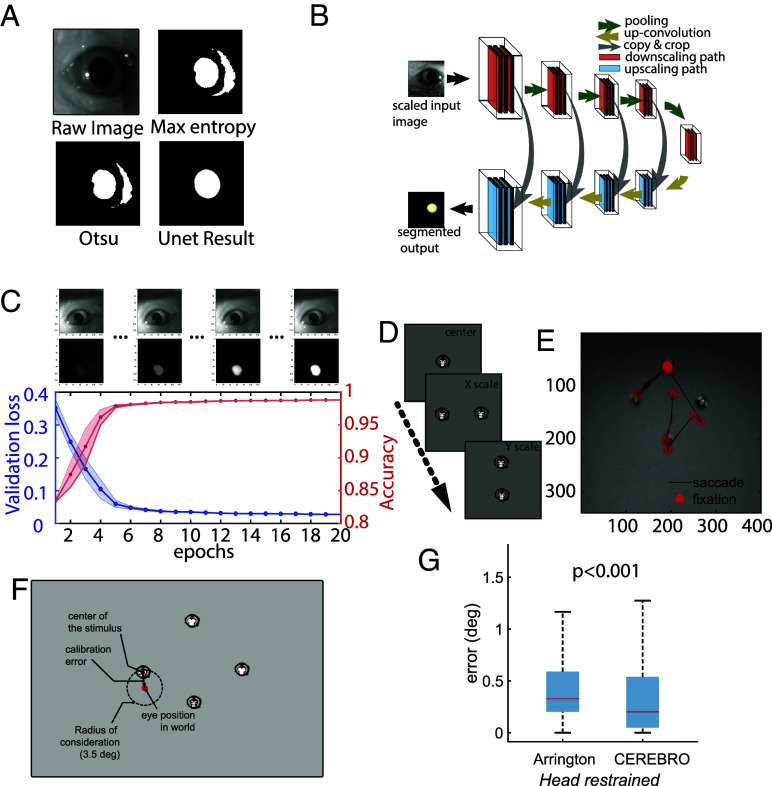
Iris/Pupil detection using segmentation Artificial Neural Network. (*A*) Conventional thresholding approach for pupil detection fails in scenes with variable light intensities. (*B*) Architecture of the segmentation neural network to crop out the iris. (*C*) The performance of the ANN matches closely to that of human annotation with relatively low training epochs (10 in this example) and relatively small amount of training data (~250 samples). (*D*) For calibrating the eye and world camera, the animals are presented with marmoset faces on a 120 Hz Liquid Crystal Display (LCD) monitor in a predetermined layout. (*E*) The calibrated eye movements can be overlaid on the world scene to determine the visual scene falling on the retina of the animal. (*F*) An illustration of the error estimation algorithm. (*G*) Accuracy comparison between Arrington eye tracker system and CEREBRO.

In traditional primate vision experiments, animals are head-fixed, and controlled infrared lighting setup allows for precise eye tracking using threshold-based pupil detection (13–15). A major challenge in tracking eye movements in freely moving animals is the lack of control over illumination, as lighting and shadows constantly change in natural environments. This point is illustrated by our application of thresholding for pupil detection of a freely moving marmoset (Fig. 2*A*), which can lead to labeling of shadows at the edge of the eye rather than the pupil. To achieve accurate primate eye-tracking in natural, freely moving conditions, an alternative method for pupil detection was needed.

Artificial Neural Networks (ANNs) offer a robust solution for pupil detection in real-world conditions, as they rely on image features rather than grayscale values, making them less sensitive to brightness changes and shadows. Such approaches have been successfully applied using commercial software (e.g., DeepLabCut), to track the pupil of freely moving mice (16). To further optimize pupil tracking in freely moving marmosets, here we trained a custom semantic segmentation ANN called UNet (Fig. 2*B*) (20) that yielded superior performance in these conditions. This workflow allows to detect pupil features robust from lighting and noise (Fig. 2*B*). With a fully trained network, we reliably detected pupils across various sessions and different animals. The network is easily trainable with fewer epochs for marmoset eyes and a training dataset of only 250 to 500 images (Fig. 2*C*). The data preparation, training of the ANN, postprocessing, and the use of a user-friendly GUI are explained in detail in SI Appendix, Fig. S3*B* and Supplementary information 6: Segmentation Artificial Neural Network for Pupil Detection. This UNet approach closely follows an architecture used previously to segment the pupil from images of human eyes [e.g., RITnet (21)]. A comparison of our UNet to the RITnet model (SI Appendix, Fig. S4) revealed that the UNet performed better at identifying the pupil from marmoset eye images, including novel views that varied in position and lighting, but was less robust on human eye images, which is consistent with the respective emphasis in the two models’ training sets. Future modifications of UNet could include additional architecture features of the RITnet model to potentially improve performance.

Accurately calculating the gaze point from the world camera necessitates that the pupil position from the eye camera be calibrated to the real-world position. To this end, we developed the following procedure. The animal is chaired and head-fixed in front of a computer screen placed ~35 cm directly in front of the subject. Because marmosets naturally look at faces (11), 1 to 12 small marmoset faces are presented as calibration targets at multiple locations on the display monitor (Fig. 2*D*). A custom-designed graphical user interface was used to adjust for scaling and offset in horizontal and vertical axes by a human operator offline. This allowed us to reliably map pupil eye position to screen coordinates (Fig. 2*E*), which when head-free, generalized to world coordinates in front of the marmoset. The GUI for pupil detection can be found at https://github.com/Vickey17/UNET_implementation_V2. To compare the system against other pupil-based eye trackers we computed the rms stability of eye position during stable fixation epochs when animals were head-fixed. We find that across seven recording sessions the rms stability was 0.05° (±0.0012 SD). These estimates provide a lower bound on the system precision that rivals head-stabilized pupil-based eye trackers such as Arrington eye trackers. To estimate our accuracy with respect to the visual stimuli on the world camera, we created an error estimation algorithm (SI Appendix, Supplementary information 8: Error Estimation for Eye in World Calibration) wherein we measure the distance between the calibrated eye position and the visual stimulus (a marmoset face; Fig. 2*F*). For comparing CEREBRO with the Arrington eye tracker, subjects were head-restrained and presented with marmoset faces on a monitor placed 35 cm from the animal. We observed that CEREBRO had significantly better accuracy as compared to the Arrington eye tracker system (Fig. 2*G*).

**Fig. 3.**
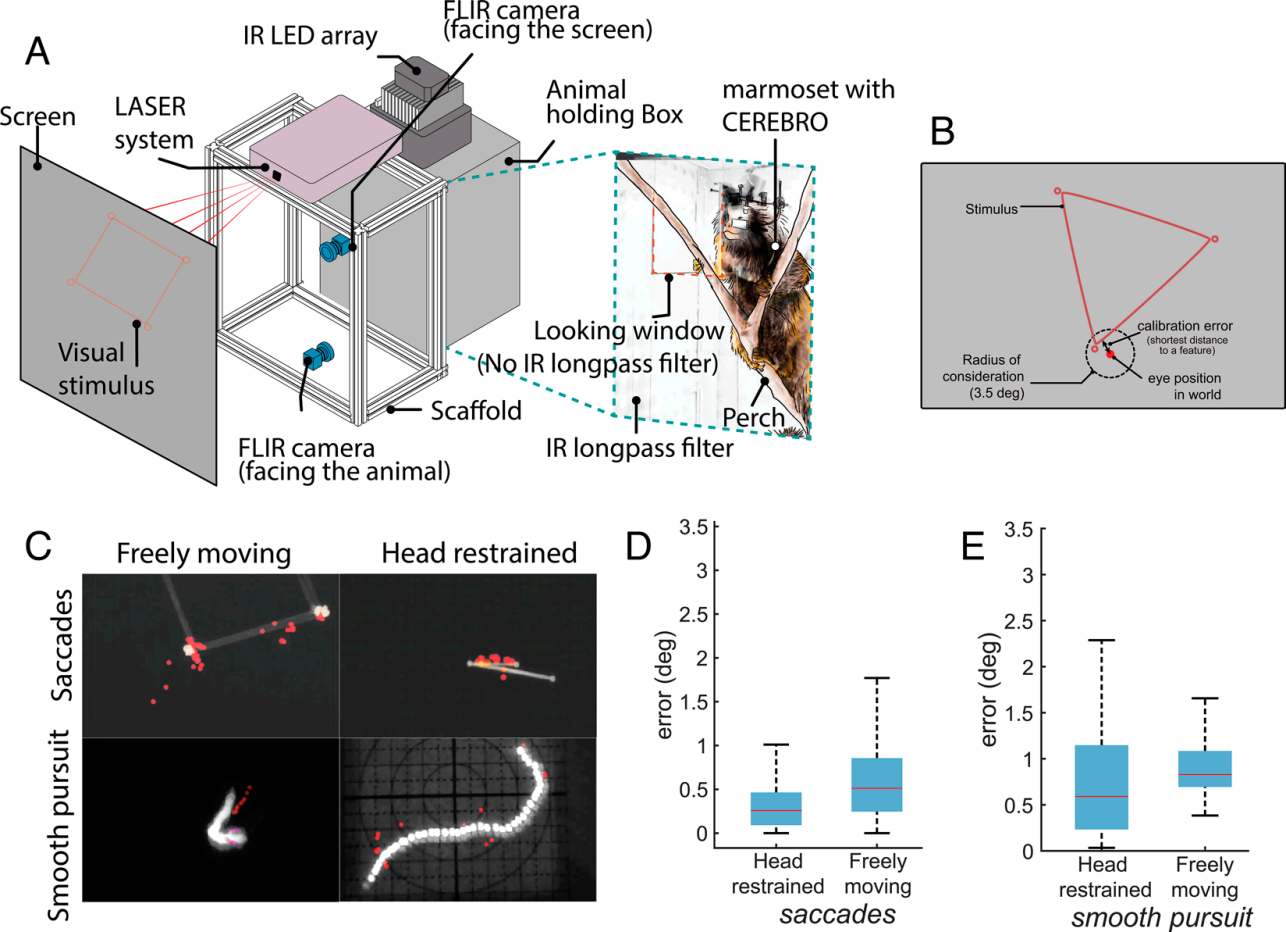
Eye/gaze accuracy in various experimental setups using CEREBRO. (*A*) Schematic for the experimental setup used to test accuracy of eye calibration in unrestrained animal. The setup consists of a dark box with a perch. The front of the box is covered with an IR longapss filter that does not allow visible light to pass except for a small window shown with a red dashed line in the *Inset* image. A galvanometric LASER system draws different geometric shapes on a screen for the animal to observe. (*B*) Schematic illustrating error estimation algorithm for freely moving eye calibration. (*C*) Maximum intensity projection of stimulus and eye in world position (red markers) for saccades and smooth pursuit trials in freely moving and head-restrained setup. (*D*) Accuracy comparison for eye calibration error for head restrained and freely moving animals during saccade trials and (*E*) smooth pursuit trails.

To estimate the accuracy of eye/gaze position in a freely moving animal, we designed a separate paradigm (Fig. 3*A*). Details of the setup are described in SI Appendix, Supplementary information 9: Freely Moving Eye Calibration Setup. Briefly, the testing arena consists of a plexiglass box with a perch for the animals to sit on. All sides of the box are painted with black acrylic to make it completely opaque except the front panel which is covered with an IR dichroic filter that allows IR light to pass for video recording of the animal inside while blocking the visible light. A small window (10 × 10 cm) is cut in the filter to allow the animal visual access to the screen outside. While the CEREBRO equipped animals sit in the dark, a top-mounted galvanometric LASER system draws geometric lines and points on a screen placed ~75 cm from the box. Each shape is drawn for a duration of 3 s followed by a dark period of 3 s. The LASER draws subject’s gaze to these shapes, thereby allowing an estimate of the eye target calibration and its error. In a second set of trials, we also used a single point that moved slowly (<5 visual degrees per second) along random trajectories. In either set of saccade or smooth pursuit trials, the gaze is projected onto the coordinates of the screen through the world view camera and error computed as the nearest distance to a point or line within 3.5 visual degree maximum bound to register looking at the feature (Fig. 3*B*). Projected eye position was found to track geometric shapes defined by points and lines (Fig. 3 *C*, *Top*) and during pursuit of the point (Fig. 3 *C*, *Bottom*). To compare accuracy against the head-restrained preparation we presented the same stimuli to head-fixed marmosets. We observe that errors cluster below two visual degrees in all conditions, well away the maximum bound for registering looking at a feature, and the median accuracy remained less than 1° in both saccade trials for geometric shapes (Fig. 3*D*) and smooth pursuit trials for a moving point (Fig. 3*E*). The median error was higher for both trial types in the freely moving compared to head-restrained condition with an increase in error by 1.98 times for saccade trials and 1.4 times for pursuit trials (Wilcoxon rank sum test, *P* < 0.0001 saccade; *P* = 0.006 smooth pursuit), but still remained under one visual degree, validating an upper bound of the system’s accuracy.

### Electrophysiology in Combination with CEREBRO.

CEREBRO was designed explicitly to create a tool to investigate the neurobiology of natural, active vision in freely moving monkeys. As such, it was designed to simultaneously record neural activity, eye behavior, and the visual scene of the animal. We tested the validity and sufficiency of the system to this end by performing experiments with CEREBRO while activity of single neurons was recorded with chronically implanted multielectrode arrays (N-form, Modular Bionics) in the primary visual cortex (V1) of the marmosets. These experiments sought to a) estimate visual tuning properties of neurons in the primate visual cortex, i.e., receptive field mapping, as well as orientation and spatial frequency tuning (22–25), using CEREBRO in more traditional head-fixed paradigms, so as to demonstrate the accuracy of our eye-tracking system by replicating these classic effects, and b) obtain eye, head, and body behavior, visual scene, and activity of single neurons simultaneously in freely moving paradigm to demonstrate the capacity of CEREBRO in investigating primate active vision.

**Fig. 4.**
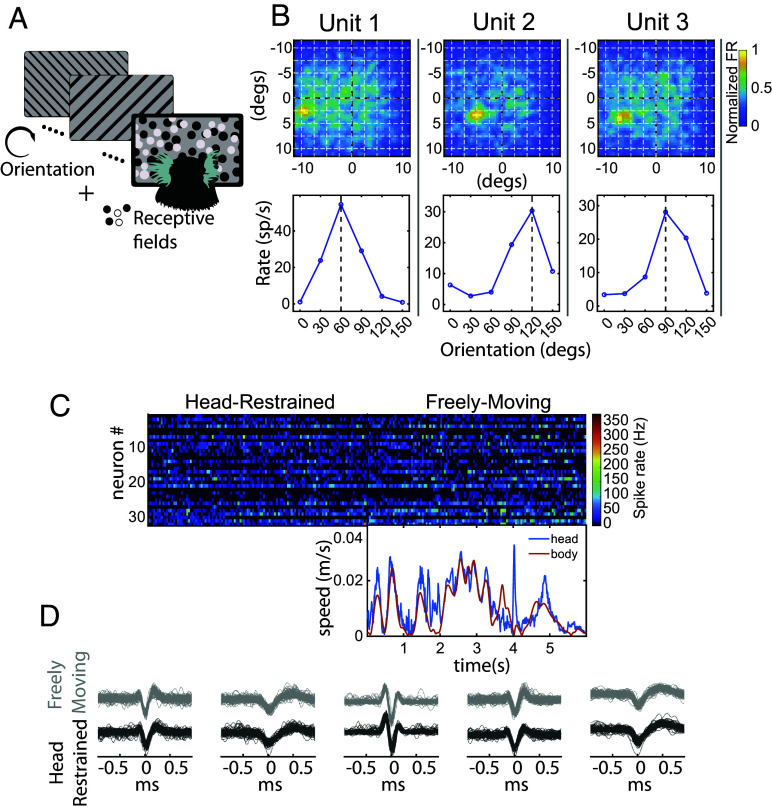
Eye tracking coupled electrophysiology in freely moving marmosets. (*A*) To validate use of CEREBRO with electrophysiology, subjects were presented with orientation grating and receptive field stimuli in a classical head fixed preparation. (*B*) The receptive field (*Top*) and orientation tuning curve (*Bottom*) is shown for three representative neurons recorded in marmoset V1 while using CEREBRO. (*C*) Example raster of 30+ single neurons in marmoset V1 while subjects are head-restrained (*Left*) and freely moving (*Right*) while wearing CEREBRO. The head (blue) and body (purple) speed of the marmoset using CEREBRO is plotted below the freely moving raster. (*D*) Example spike waveforms from five exemplar neurons demonstrate that we were able to stably record from same neurons in head-restraint vs. freely moving conditions.

To recapitulate the tuning properties of V1 neurons, subjects were head-fixed while wearing CEREBRO and presented with the following stimuli: flashing dots for receptive field mapping, and drifting gratings with different orientations and spatial frequencies for tuning properties (Fig. 4*A* and SI Appendix, Supplementary information 10: Receptive Field and Tuning Property of V1 Neurons). Critically, marmosets were allowed to free-view the video screen during stimulus presentation and offline corrections for eye position enabled accurate reconstruction of visual properties following a recently developed free-viewing approach (26). A key difference here from the previous study with head-fixed marmosets is that the visual input is obtained by the world camera’s view along with the estimated eye position from CEREBRO instead of what was known to be displayed on the screen, thus validating that these methods could generalize to real-world stimuli. Results from three example neurons demonstrate visual receptive fields estimated at the peak visual latency (Fig. 4 *B*, *Top* row) and the orientation tuning at the peak visual latency (Fig. 4 *B*, *Bottom* row) estimated from the CEREBRO world camera images. These results demonstrate that our eye-tracking system and calibration approach can accurately record neural activity in response to visual stimuli on the primate retina.

Subjects were also allowed to actively explore a 200 cm × 100 cm arena decorated with various visual stimuli. Activity of single neurons was continuously recorded in the period of head-restraint and freely moving condition; body and head movements were simultaneously recorded using *OptiTrack* system (Fig. 4*C*, Online Methods, Head and Body Movement Tracking Using OptiTrack). The comparison of spike waveforms in head-restraint vs. freely moving condition among five example neurons demonstrates the stability of neural recording throughout the session (Fig. 4*D*). Within the same session, the eye behavior and the neural activity (spike rate) change between the head restrained vs. freely moving animal.

### Visual Behavior of Freely Moving Marmosets.

**Fig. 5.**
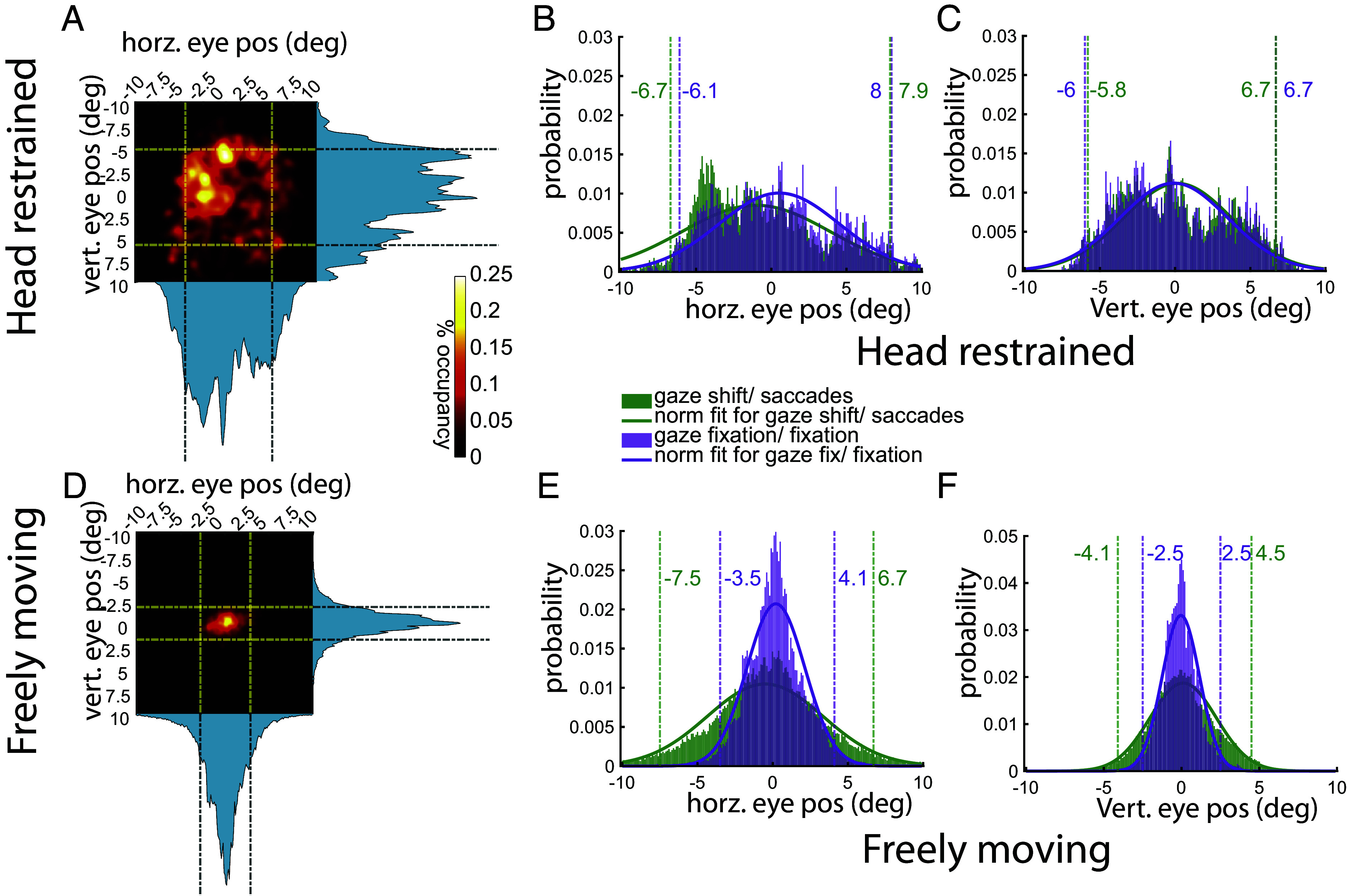
Characteristics of eye/gaze behavior in various experimental setups using CEREBRO. (*A*) Heatmap of eye position in a restrained marmoset with dashed lines marking 2 SD mark. (*B* and *C*) distribution of horizontal and vertical eye position split by fixation (purple) vs. saccade (green) with corresponding dashed lines representing 2 SD of data variation. (*D*) Heatmap of eye position in an unrestrained marmoset. (*E* and *F*) Distribution of horizontal and vertical eye position split by gaze fixation (purple) and gaze shifts (green). Color-matched dashed lines represent 2 SD of variation in data.

**Fig. 6.**
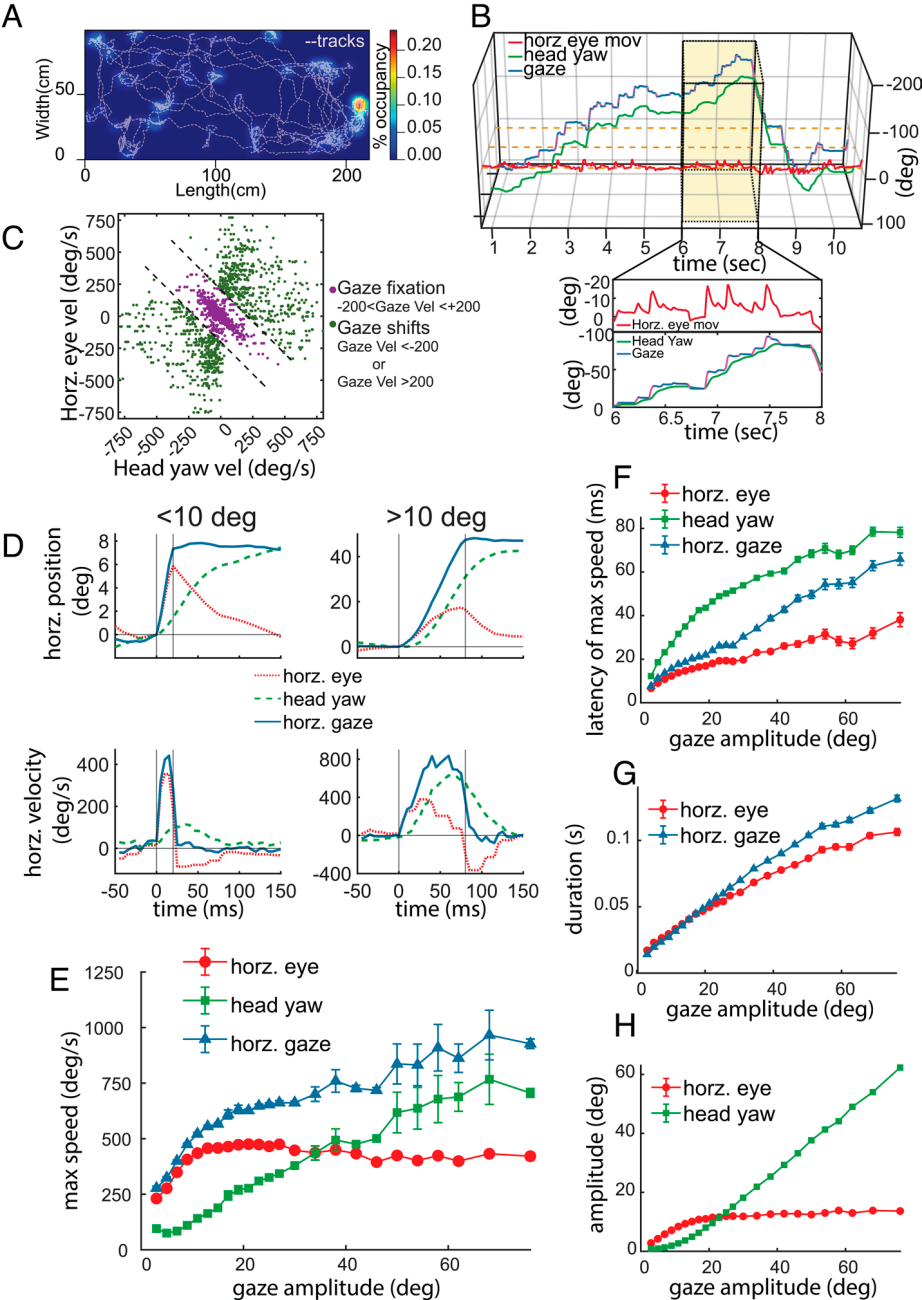
Gaze characteristics in freely moving marmosets. (*A*) Representative session of activity pattern for marmosets in an open arena (200 cm × 100 cm × 240 cm). Gray dotted lines indicate periods of locomotion, while heat maps plot stationary occupancy. (*B*) Plots the estimate of the animal’s gaze (blue) with CEREBRO. Horizontal eye movement (red) and head yaw (green) are also plotted. *Inset* magnifies a 2 s period of time. (*C*) Horizontal eye movement and head yaw in freely moving marmosets are shown. Green dots indicate gaze fixations, while purple dots indicate gaze shifts. (*D*–*H*) Eye movements (red), head movements (green), and gaze shifts (red) in freely moving marmosets. (*D*) Plots the position (*Top*) and velocity (*Bottom*) of eye movements, head movements and gaze shifts <10° (*Left*), and >10° (*Right*). (*E*) Shows the maximum speed and gaze amplitude. Plots the latency to max speed (*F*), duration (*G*), and amplitude (*H*) as a function of gaze amplitude.

We compared the eye behavior of a head-restrained marmoset and a freely moving marmoset. In the head-restrained context, the animal was presented with a series of naturalistic images. During the freely moving context, the animal was placed in an open arena and allowed to explore the environment. Consistent with previous studies (11), analysis in the head-restrained context revealed that the eye in head position i.e. the horizontal and vertical eye position spanned ±5 visual degrees for 2 SD of all the positions (Fig. 5*A*) and the eye position distribution for saccades vs. fixation were identical in this case (Fig. 5 *B* and *C*). However, the results for freely moving marmoset are starkly different. First, the eye in head position is much more limited in a freely moving animal spanning only ±2.5 visual degrees (Fig. 5*D*). Moreover, most of the eye positions at the edges of the range were involved in gaze shifts and not gaze fixations (Fig. 5 *E* and *F*). These findings suggest that during stable epochs of gaze, the head position provides a reliable estimate of gaze position. The primary motivation to develop CEREBRO was to precisely quantify the characteristics of eye movements and gaze in freely moving, naturally behaving primates. To this end, we recorded visual behavior in marmosets wearing CEREBRO as they explored an open rectangular arena (Fig. 6*A*). As marmosets do not continuously move when in open-field environments, we distinguished between the following two behavioral states: a) “stationary”—the monkey was seated or standing and visually scanning the environment without physically changing locations b) “locomotion”—the monkey was physically moving and changing its position in the environment. Marmosets typically remained stationary for extended periods at fixed locations (occupancy map in Fig. 6*A*), and then moved between those locations (gray traces, Fig. 6*A*). We conjectured that the gaze dynamics likely differs between these two behavioral states, as each differs in motoric demands and exploratory function.

In a freely moving primate, visual exploration is accomplished by changing gaze which can be defined as a sum of head and eye movements (Fig. 6*B*). Stable eye positions in this context are rare because even when an animal fixates at a fixed position in the scene—referred to here as a gaze fixation—the eyes must still move smoothly to compensate for any head movements for retinal stabilization. These compensatory eye movements reflect the vestibular ocular reflex (VOR) that well conserved across species and normally engaged to reduce retinal motion that is due to head and body motion (8, 9, 27–29). These compensatory movements correlate negatively with the head-movement velocity to subtract its effect and achieve stable gaze. By contrast, during rapid gaze shifts, equivalent to traditional saccades in the head-fixed case, the VOR is suppressed and there is a combination of conjugate head and eye movements along the same direction, with eye velocity reversing at the end of the rapid shift as VOR is restored and gaze is again stabilized by compensatory eye movements compensating for continuing head velocity. In our current study, we have primarily focused on the horizontal eye movements and head yaw since it has been demonstrated that horizontal pursuit eye movements are more accurate and symmetric than vertical ones in primates (30, 31). The *Inset* in Fig. 6*B* illustrates this point as the sum of eye and head position (gaze) exhibits steps in position with stable periods in between. While head position is continuously changing, the eye position exhibits saw-toothed type patterns in which a jump in position is followed by decay backward that compensates for the change in head position. To distinguish epochs of rapid gaze shifts and compensatory movements, we set a gaze velocity threshold of ±200°/s. Examination of eye movements during gaze shifts and gaze fixations revealed a clear trend of negative correlation between head and eye movements for fixation periods reflecting compensatory eye movements to stabilize gaze (purple, Fig. 6*C*) in a freely moving marmoset. For the gaze velocity threshold set, we find compensatory movements are well separated from other rapid gaze shifts (green, Fig. 6*C*).

A core feature of mammalian ocular-motor behavior is the main sequence; a characteristic linear relation between the amplitude and peak velocity of eye movements. We quantified this relationship here to test whether the main sequence is evident in a freely moving primate using CEREBRO. For conjugate gaze shifts we observed a characteristic pattern wherein eye velocity reached a peak velocity more quickly than head-velocity. As head-velocity decayed with a long tail, eye velocity reversed direction in order to counteract the head-velocity and stabilize gaze (Fig. 6*D*). This pattern was evident both for small and large gaze shifts, with the duration of the gaze shift being longer for larger gaze shifts. To quantify these patterns more accurately, we next plotted the peak velocity, latency to peak, and duration of the eye, head, and gaze components as a function of gaze amplitude (Fig. 6 *E*–*H*). Although the main sequence was evident in freely moving marmosets here, evidence suggested differences in how the eye and head components contribute to it as a function of gaze amplitude (Fig. 6*E*). Whereas the peak velocity of eye movements saturated for gaze shifts of roughly 10° in amplitude around 400°/s, the peak velocity of head shifts continued to increase even for the largest measured shifts out to 80°. The latency of the peak eye velocity always leads peak head velocity as a function of gaze amplitude with each growing as a function of shift amplitude (Fig. 6*F*). The peak of the gaze velocity follows more closely with eye velocity for small shifts (<36°) and with head-velocity for larger shifts. The duration of gaze shifts follows a roughly linear relation with gaze amplitude (Fig. 6*G*). In summary, the components of eye and head shifts differ in their contribution to gaze shifts depending on the amplitude of the shift, with eye velocity rapidly saturating in its maximum velocity for shifts of about 10° in size after which the slower initiating head-shift contributes more to the total gaze movement. As the gaze amplitude becomes larger, the contributions of the head increase linearly to the overall shifts while the eye saturates in its contribution after about 20 visual degrees (Fig. 6*H*).

**Fig. 7.**
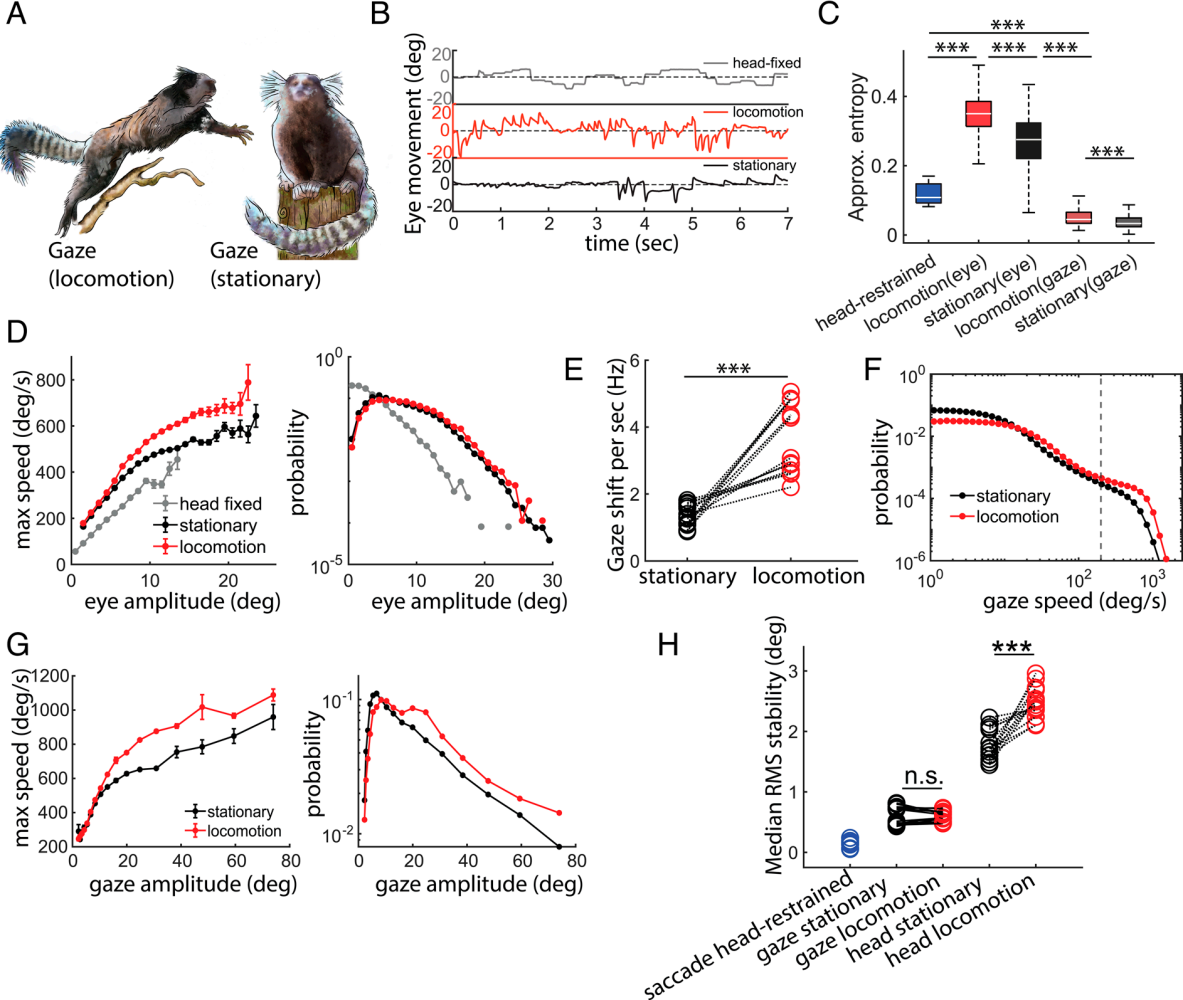
Characteristics of horizontal eye movements in freely moving marmosets. (*A*) An illustration of the gaze shifts in a freely behaving marmoset in two different states of locomotion (*Left*) and stationary (*Right*). (*B*) Animals exhibited noticeably different eye behavior in various contexts i.e. chaired, locomotion, and stationary. (*C*) Horz. eye movements have significantly less “approximate entropy” in head-fixed context as compared to freely moving. Although horz. gaze has the least approximate entropy of all the contexts. This shows that a freely moving gaze is computationally more regular than a head-fixed context. (*D*) Freely moving animal exhibits larger range of horz. eye amplitude and the speed of horz. eye movements is higher during locomotion than stationary state. Marmosets make more gaze shifts (*E*) during locomotion with higher speed (*F*) and larger amplitudes (*G*). (*H*) rms stabilization of head (yaw) and horz. gaze shows that despite poorer stability because of head movements, gaze remains remarkably stable during locomotion and stationary epochs.

To determine how visual behavior differed between behavioral contexts we compared the eye movements of marmosets when head-fixed and freely moving, distinguishing between instances when individuals were visually scanning the environment while “stationary” and instances when animals were actively moving through the world by “locomotion” in the latter context (Fig. 7*A*). As demonstrated in Fig. 7*B*, differences in marmosets’ visual behavior when head-fixed and both freely moving contexts were stark, with the eye movements being notably dynamic in freely moving contexts reflecting VOR adjustments for self-motion. We quantified these differences further by calculating the approximate entropy of marmoset eye movements in each of these three contexts (32). This analysis estimates the randomness of a time series where higher value of approximate entropy means a system that is more random and vice versa—thereby allowing a metric for quantification of the visual behavior as a time series. Overall, we observed that marmoset eye movements in the head-restrained condition exhibited a significantly lower entropy than both the freely moving conditions (stationary and locomotion; Fig. 7*C*), suggesting that eye movements in a freely moving animal is more chaotic/random. However, the approximate entropy for “gaze” of freely moving animals—the combination of both head and eye movements—a different pattern emerged. Here, the approximate entropy in both freely moving contexts was in fact lower than in the head-fixed context. This suggests that the synergistic combination of head and eye movements while freely moving yields a less random/chaotic scanning of the visual environment than when head-fixed, a likely computational optimization of the visual system to sample the scene in animals as they naturally move and explore the world populated with elements with varying salience. The statistics of maximal speed and amplitude of the eye movement shows a significant faster and larger eye movement in the freely moving context (Fig. 7*D*).

In addition to significant contrasts in visual behavior between head-fixed and freely moving contexts, we also observed differences in the latter when marmosets were stationary or locomoting. Specifically, marmosets make significantly more gaze shifts per second (Fig. 7*E*) with greater speeds (Fig. 7*F*) and larger amplitudes and maximal speed (Fig. 7*G*) during locomotion than during stationary phases. This pattern raises the question whether the gaze of the animal is less stable during locomotion given the larger gaze shift amplitudes. To investigate that, we calculated the rms stabilization of the gaze during gaze fixations during locomotion and stationary contexts (16). We use rms as a measure to quantify the deviation of gaze and head yaw during stabilization periods (gaze fixations). A smaller rms during fixation epochs indicates a more stable fixation while a higher value for rms will indicate poor stabilization. Analyses revealed that gaze was on average about three times more stable than head movements during gaze fixations (Fig. 7*H*). When comparing the stability of gaze to that of the head, however, we observed that head movements were less stable during locomotion gaze stability but surprisingly remained similar during locomotion (Fig. 7*H*), despite the increase in head-motion. Thus, while the head is clearly less stable during locomotion, compensatory eye movements appear to provide better stabilization achieving stable epochs of gaze fixation during locomotion. This highlights the importance and potential context dependence of gaze stabilization, wherein it appears to be enhanced to achieve stability during locomotion.

## Discussion

We developed an innovative head-mounted eye-tracking system—CEREBRO—to quantify the visual behavior of freely moving primates, specifically common marmosets (~300 to 400 g). Our system records eye movements at 90 FPS and world scene at 63 FPS, while also overcoming challenges in pupil detection caused by changing lighting through a segmentation neural network. CEREBRO also integrates with wireless neural recording to expand its application to studies investigating the supporting neural processes of visual behavior. While findings reported here in freely moving primates recapitulate the core features of conjugate gaze movements from prior studies using head-free—but chair-restrained—macaques (33, 34) and extend those to freely moving primates, analyses also revealed several related characteristics of visual behavior that have not been reported previously. Perhaps most notably, we observed that the entropy of the gaze behavior was significantly lower when animals were freely moving than when head fixed, and that the stability of the visual gaze remains unchanged during locomotion. CEREBRO enables the study of active primate visual processing in natural, freely moving contexts, highlighting the importance of coordinated head-eye movements for stabilizing vision during naturalistic exploration.

CEREBRO is designed to allow for studying visual behavior coupled with neural responses in a freely moving animal. To that end, we tested our system during neural recordings to characterize orientation tuning and receptive fields using classical reverse correlation methods during head-fixation. As demonstrated in Fig. 4, we were able to recover clear receptive fields and orientation tuning of V1/V2 neurons using eye tracking from CEREBRO with the view from the world camera. In the future, this system will more broadly enable us to investigate the role of saccade related modulation in head-fixed vs. head-free contexts, and more so, to obtain receptive fields in freely moving animals. Our estimates of gaze accuracy, though less accurate in the free-moving than head-fixed conditions (Fig. 3 *D* and *E*), are on median under one visual degree. Thus, reconstruction of visual receptive fields, as was shown for the head-fixed condition using the scene camera (Fig. 4*B*), should also be feasible for free-moving cases given similar flashed visual stimuli. However, a key challenge during free-motion is that the statistics of the visual input are radically different and instead controlled by the animal, which provides insights into active vision but also poses quantitative challenges for fitting receptive field models due to the spatiotemporal correlations found during natural motion and with real visual scenes. These issues can be addressed by exploiting computational approaches that are highly data-efficient. Indeed, the receptive field mapping of V1 neurons in freely moving mice, where visual receptive fields are much larger than primates, has been achieved with a Generalized Linear Model approach (3). Similar methods could be applied in free-moving marmosets, especially if the visual areas under study have receptive field sizes larger than gaze tracking error, as in the peripheral visual field. Furthermore, the video streams from our world camera will provide statistics for how the visual scene and optic flow changes during free motion and help to expand our understanding of how natural scene statistics are processed in the visual cortex.

The contribution of eye and head movements toward conjugate gaze shifts in freely moving marmosets were qualitatively similar that of chair restrained but head-free macaques and other mammals (7, 33, 35–39). However, marmosets did exhibit some measurable differences relative to macaques. For example, marmoset eye movement velocity and amplitude saturate for much smaller gaze shifts around 10 to 20°, after which the head contributes to the bulk of the shift in gaze (Fig. 4*H*), whereas in the macaque this transition does not occur until much larger gaze shifts between 20 to 40° (33). Moreover, most gaze shifts in macaques under 20° in size are predominantly driven by shifts in eye position and not the head, while in the marmoset the same gaze shifts would have significant head-movement components. Only the smallest of gaze shifts under 5 to 10° in the marmoset, a range comparable to the movements made in head-fixed marmosets (Fig. 6*D*), are dominated by changes in eye position after which the head would normally make significant contributions. These differences likely reflect an efficiency tradeoff related to smaller head-size in marmosets (40).

Because experiments were performed in head-fixed and freely moving conditions, this dataset can be compared to prior studies and extend our understanding of visual behavior in natural contexts. Marmoset oculomotor range is relatively fixed within about 10 visual degrees when head-fixed, as reported previously (11), but that range increases to ~20° (Fig. 7*D*) when individuals were freely moving suggesting the limited oculomotor range when head-fixed represents more of a motor preference than a physical limitation. In a previous study of chaired but head-free marmosets, a paradigm was used to evoke large gaze shifts involving up to 180° head rotation (12). Although similar large shifts were rare in the study here, a similar linear relation between peak head velocity and gaze shifts that peak near a velocity of 750°/s for an 80° gaze shifts were evident in freely moving marmosets suggesting that this aspect of head-gaze control are relatively invariant to the form of the task. A direct comparison of the two freely moving contexts here revealed notable differences including larger amplitude gaze shifts with higher maximum velocities when locomoting (Fig. 7 *D*–*G*). By contrast, when stationary and visually scanning the environment, marmoset biased to smaller gaze shifts indicating that motor demands of actively moving through space likely drives differences in how the head and eyes coordinate to stabilize the visual field.

Quantifying primate visual behavior with the innovative eye-tracking system here afforded the powerful opportunity to examine whether our assumptions about natural vision based on studies in head-restrained animals are accurate. While qualitative similarities in features of conjugate eye movements between head-fixed and freely moving contexts were evident, it was also apparent that certain assumptions about results in the more conventional paradigm may not be strictly true. The first being that gaze stability did not worsen despite greater eye and head movements during locomotion. We also observed that the approximate entropy of the marmoset visual system was significantly lower (i.e., improved) when individuals were freely moving than head fixed suggesting that the collective visual system is more consistent and predictable when marmosets are moving naturally. Likewise, a comparison of freely moving marmosets when stationary—i.e., visually scanning—and locomoting revealed that the stability of visual gaze—as measured by rms—was significantly better when animals were locomoting. In other words, despite an increase in the number, speed, and length of the gaze shifts when locomoting, vision actually became more stable. These findings show that the coordinated movements of head and eyes have been optimized to accommodate self-motion in a diversity of ethological contexts.

The context dependence of visual stability between the stationary scanning and locomotion states could reflect changes in VOR at the neural level, as well as other positional strategies that optimize the VOR system (41, 42). Such context dependence of gaze control has long been appreciated from head-mounted eye-tracking studies in humans (43, 44), and recently includes free motion in natural contexts (45). The technical parallels between these eye-tracking systems in human experiments and CEREBRO allows for comparisons between these primate species, as well other taxa. In humans, for example, the gain for angular VOR is a function of locomotion speed (46, 47). Those findings are consistent with the rms stabilization of gaze in marmosets being maintained, or even slightly improved, during locomotion as compared to when they are stationary and scanning the scene (Fig. 7*H*). An increase in VOR gain during locomotion would explain how stability is maintained even though head and gaze movements increase in frequency and amplitude during locomotion (Fig. 7 *E*–*G*). Further, we measured the negative correlation between horizontal head and eye velocity during periods of stable fixation to estimate the VOR gain directly (SI Appendix, Supplementary information 11: Gain in VOR during Locomotion). We find that VOR gain increases during locomotion as compared to stationary scanning, consistent with prior human studies and the maintenance of rms stability during locomotion.

Another parallel across humans and marmosets is the change in the relative contribution of head and eye movements to gaze shifts with increasing gaze amplitude (48). We found that larger head amplitudes lead to less eye contribution (Fig. 6*H*) wherein the eye amplitude saturates at ~10° and then the putative target is achieved with help of head movement. In humans the reliance on head movements does not typically occur until much larger gaze shifts are required, but this also can depend critically on the task context, including the speed of locomotion (46). The natural movement statistics of head position in free-moving primates, however, differs substantially from rodents (49), suggesting that at least some mechanisms for visual stabilizations may not be evident across all vertebrates or mammals. In future studies, CEREBRO can be used to address other features of oculomotor control relevant to free-motion in primates and other taxa, including vergence and torsional eye movements (50, 51). The current eye-tracking system offers an opportunity to study how computational constraints and movement strategies during active vision act to achieve stability and provide high acuity vision in different contexts.

CEREBRO was explicitly designed to integrate studying visual behavior with neural responses in freely moving animals. To that end, we tested our system on classical visual correlates like orientation tuning and receptive fields to assess the quality of responses collected by our system with the conventional methods. As demonstrated in our findings (Fig. 4), we obtained clear receptive fields and orientation tuning of V1/V2 neurons using just the eye and the world camera of our system. However, quantifying the visual information at the level of the retina and its representation in the early visual cortex presents particular analytic challenges beyond the scope of this study. In contrast to head-fixed paradigms in which controlled visual stimuli can be presented to map receptive fields, the natural environment has highly correlated spatiotemporal features in the visual input, as well as low contrast in a large number of images (e.g., when the animal looks at the ceiling or the floor). These issues cause insufficient statistical power in the collected samples and require more advanced computational approaches to resolve that were beyond the scope of this study. Fortunately, an elegant experiment in mice (3) provides a roadmap that we will pursue in future studies. Future experiments will also address other fundamental questions such as dissecting the role of visual flow on the neural population to understand how optical flow aids in separating foreground and background elements as observed in humans (52). By eliminating a critical bottleneck, CEREBRO opens the door to studies of natural active vision and its supporting neural mechanisms in primates that were not possible before.

Despite its potential, CEREBRO is not without its limitations. For example, marmosets are an arboreal species and face challenges in a 3D context that differ from the 2D conditions tested here. It is, therefore, unclear how the wearable technology might affect their movements in such environments, and by extension the data and our interpretations of those data. Likewise, one of our key plans for future research is to leverage this system to investigate social perception and its underlying neural mechanisms (e.g., face patches) but it is uncertain whether the headgear might influence social interactions, potentially biasing the data. Finally, we have made significant efforts to quantify the precision of the marmoset gaze targets in a freely moving context, but it is important to recognize that these are estimations. Like all eye-tracking systems, real-world complexity and variability has the potential to introduce more error in our quantification as compared to more controlled settings.

Among the most significant selective forces acting on animals over evolution is to move and interact with the world, and to do so requires sensory feedback. The animals’ behavior can be thought of as an outcome filtered by the environment and the animals’ potential actions (53). For a freely moving animal, this “affordance landscape” can be very dynamic given the exteroceptive (sensory feedback), proprioceptive, and interoceptive feedback. It has been argued that perception is not just about creating an internal representation of the world based on sensory inputs, but instead filters responses relevant to the environment and animals’ internal state (54). Despite this, the study of the primate visual system has largely ignored considerations of movement and relationships between the sensory inputs and affordances. Head-restrained experiments limit the sensorimotor and affordance landscape of an animal that potentially bias results in a way that differs from naturalistic behaviors in which an animal acts as an agent. Freely moving animals express continuous behaviors based on the elements in the environment, history of previous actions and internal state which is starkly different from a trial-based structure and lead to many interesting discoveries about computation strategies in the brain. Leveraging our innovative head-mounted eye-tracking system for marmosets, we provide compelling evidence that the coordinated actions of eye and head movements are optimized to stabilize primate vision and increase its predictability. These patterns emphasize the significance in considering the ethological relevance of how primates are tested in vision studies. Evidence suggests that neural responses in head-fixed paradigms are not necessarily predictive of how the same single neurons—or population ensembles—respond in naturalistic contexts (55–57). Indeed, investigations of vision in freely moving mice suggest that at least some elements of neural activity in this context are distinct (3, 58). Our innovative eye-tracking system can be leveraged in marmosets to precisely examine the primate visual system during natural, freely moving behaviors (5, 59) and address a suite of foundational questions that we have as of yet been unable to investigate with sufficient quantitative rigor.

## Materials and Methods

### Experimental Model and Study Participant Details.

Experiments described here involved two 2yo common marmosets (monkey M, male; monkey S, female). Monkey M had a chronic implant in left V1 and monkey S had bilateral chronic implants in V1. All surgeries and experiments were approved by the University of California, San Diego, Institutional Animal Care and Use Committee (IACUC) in accordance with National Institute of Health standards for care and use of laboratory animals.

### Fitting the Animal with CEREBRO.

The head assembly for CEREBRO is custom fitted to every animal. For the initial fitting, the animal is anesthetized using a combination of Ketamine (dose: 20 mg/kg) and Acepromazine (dose: 0.75 mg/kg). All the parts (SI Appendix, Fig. S1*B*) are adjusted for best view of the eye and the front camera and fastened using set screws.

### Multiple System Synchronization.

Our Neural recording systems, motion trackers, and CEREBRO were all synchronized using an ESP32 microcontroller based custom board. Additional details of the synchronization protocols are described in SI Appendix, Supplementary information 12: Synchronization of Various Systems Using ESP32 Microcontroller.

### Electrophysiology.

Neural activity was recorded with 64 channel N-form arrays (Modular Bionics, Berkeley, CA) chronically implanted in V1 with a wireless Neurologger (SpikeLog-64, Deuteron Technologies). Spike sorting was performed offline using Kilo sort (60) and manually curated using the graphic user interface Phy.

### Head and Body Movement Tracking Using OptiTrack.

Head and body movements were recorded using the OptiTrack motion tracking system (www.optitrack.com). Three IR reflective beads (12 mm dia) were placed on the animal’s head, and a single bead was added to the backpack for body movement. The arena was equipped with 10 OptiTrack cameras, calibrated to ensure the worst camera error was under 0.1 mm. The collected data were manually curated to remove false markers and gaps were filled with linear interpolation.

### Locomotion Behavior Analysis.

Locomotion was detected using body and head tracking data from OptiTrack. Locomotion was defined as epochs when the body IR bead’s velocity (after a 0.2 Hz low-pass filter) crossed a threshold of 5 cm/s. To ensure it was true locomotion, we added two conditions: the movement duration must exceed 3 s, and the head position must be below 18 cm. These conditions were verified by manually matching the data with video. Periods meeting these criteria were classified as locomotion, while the rest were considered sedentary.

## Supplementary Material

Appendix 01 (PDF)

## Data Availability

Head, eye, and body locomotion data and related code, GUI, STL designs data have been deposited in Dryad and Github (10.5061/dryad.8gtht76xb (61), https://github.com/Vickey17/UNET_implementation_V2 (62), and https://github.com/Vickey17/CEREBRO_HeadPiece (63)).
